# Melatonin-Stimulated Triacylglycerol Breakdown and Energy Turnover under Salinity Stress Contributes to the Maintenance of Plasma Membrane H^+^–ATPase Activity and K^+^/Na^+^ Homeostasis in Sweet Potato

**DOI:** 10.3389/fpls.2018.00256

**Published:** 2018-02-27

**Authors:** Yicheng Yu, Aimin Wang, Xiang Li, Meng Kou, Wenjun Wang, Xianyang Chen, Tao Xu, Mingku Zhu, Daifu Ma, Zongyun Li, Jian Sun

**Affiliations:** ^1^Institute of Integrative Plant Biology, Jiangsu Key Laboratory of Phylogenomics and Comparative Genomics, School of Life Sciences, Jiangsu Normal University, Xuzhou, China; ^2^Xuzhou Institute of Agricultural Sciences in Jiangsu Xuhuai Area, Xuzhou, China; ^3^Beijing Qiji Biotechnology Co., Ltd., Beijing, China

**Keywords:** melatonin, sweet potato, K^+/^Na^+^ homeostasis, triacylglycerol, fatty acid β-oxidation, PM H^+^–ATPase

## Abstract

Melatonin (MT) is a multifunctional molecule in animals and plants and is involved in defense against salinity stress in various plant species. In this study, MT pretreatment was simultaneously applied to the roots and leaves of sweet potato seedlings [*Ipomoea batatas* (L.) Lam.], which is an important food and industry crop worldwide, followed by treatment of 150 mM NaCl. The roles of MT in mediating K^+^/Na^+^ homeostasis and lipid metabolism in salinized sweet potato were investigated. Exogenous MT enhanced the resistance to NaCl and improved K^+^/Na^+^ homeostasis in sweet potato seedlings as indicated by the low reduced K^+^ content in tissues and low accumulation of Na^+^ content in the shoot. Electrophysiological experiments revealed that exogenous MT significantly suppressed NaCl-induced K^+^ efflux in sweet potato roots and mesophyll tissues. Further experiments showed that MT enhanced the plasma membrane (PM) H^+^–ATPase activity and intracellular adenosine triphosphate (ATP) level in the roots and leaves of salinized sweet potato. Lipidomic profiling revealed that exogenous MT completely prevented salt-induced triacylglycerol (TAG) accumulation in the leaves. In addition, MT upregulated the expression of genes related to TAG breakdown, fatty acid (FA) β-oxidation, and energy turnover. Chemical inhibition of the β-oxidation pathway led to drastic accumulation of lipid droplets in the vegetative tissues of NaCl-stressed sweet potato and simultaneously disrupted the MT-stimulated energy state, PM H^+^–ATPase activity, and K^+^/Na^+^ homeostasis. Results revealed that exogenous MT stimulated TAG breakdown, FA β-oxidation, and energy turnover under salinity conditions, thereby contributing to the maintenance of PM H^+^–ATPase activity and K^+^/Na^+^ homeostasis in sweet potato.

## Introduction

Soil salinity, a major environmental stress, disturbs normal growth, cellular ion homeostasis, and metabolic pathways in plants and decreases the crop yield by approximately 20% in irrigated lands (Shabala et al., 2016). Maintaining an appropriate cytosolic potassium–sodium ratio under high salinity condition is an important determinant of plant salt tolerance (Anschütz et al., 2014). Plants use various physiological mechanisms, including extrusion of Na^+^ to the apoplastic space, sequestration of Na^+^ in vacuoles, and control of xylem loading of Na^+^, to cope with Na^+^ toxicity (Munns and Tester, 2008; Kumari et al., 2015). Salt-tolerant species (such as halophytes) or crop varieties exhibit high K^+^ retention capacity in the root and leaf tissues (Anschütz et al., 2014; Kumari et al., 2015). Scholars have confirmed the strong positive correlation between cellular K^+^ retention and salt tolerance of the whole plant in a broad range of species, including barley (Chen et al., 2005), wheat (Cuin et al., 2008), poplar (Sun et al., 2009b), sweet potato (Yu et al., 2016), *Brassica* species (Chakraborty et al., 2016), and halophytes (Bose et al., 2015). High PM H^+^–ATPase activity under salinity condition contributes to the maintenance of low depolarized membrane potential, thereby decreasing the opening of depolarization-activated K^+^ outward-rectifying channels and K^+^ efflux triggered by high salinity (Shabala and Pottosin, 2014).

*N*-acetyl-5-methoxytryptamine (melatonin, MT), an important animal hormone, performs multiple functions as circadian rhythm regulator and antioxidant (Hardeland et al., 2012; Reiter et al., 2016). In 1995, MT was first identified in plants through high-performance liquid chromatography and radioimmunoassay analyses (Dubbels et al., 1995; Hattori et al., 1995). MT is a multifunctional molecule that regulates various physiological and molecular processes in plants. These processes include leaf senescence (Wang et al., 2013), responses to abiotic and biotic stresses (Zhang et al., 2015), postharvest physiological deterioration (Ma et al., 2016), and anthocyanin synthesis (Zhang et al., 2016). Various studies showed that MT is important in determining plant salt tolerance (Li et al., 2012; Liang et al., 2015; Shi et al., 2015; Wei et al., 2015). The beneficial role of MT on plant salt tolerance was first reported in 2012 (Li et al., 2012). Several studies revealed that endogenous MT or exogenous application of MT contributes to the improvement of K^+^/Na^+^ homeostasis in different plant species under salinity conditions (Li et al., 2012; Chen et al., 2017). In addition, exogenous application of MT stimulated the expression of genes related to K^+^ and Na^+^ transport in salinized *Malus* plants (Li et al., 2016). Furthermore, endogenous MT has been shown to play a significant role in triggering reactive oxygen species (ROS) signaling and in mediating ROS-dependent Na^+^ extrusion in salinized *Arabidopsis* plants (Chen et al., 2017). These pieces of evidence clearly showed that MT improves K^+^/Na^+^ homeostasis in plants under high salinity conditions. However, the mechanisms underlining the MT-regulated K^+^/Na^+^ homeostasis in salinized plants is still unclear.

Changes in lipid metabolism and composition and distinct lipid molecular species are associated with altered plant growth, development, and responses to environmental stresses, including salinity stress (Natera et al., 2016). The critical role of lipid remodeling in plant salt tolerance comes from the fact that lipids have a great impact not only on changing the membrane integrity, permeability and fluidity but also on modulating membrane proteins activity and cellular signaling pathway (Mansour et al., 2015). Lipidomic analysis of maize (*Zea mays*) mesophyll cell (MC) and bundle sheath cell (BSC) chloroplasts revealed a less reduction of MGDG content in salt-stressed BSC chloroplasts; this property maintained the stabilization of thylakoid membranes and rendered BSC chloroplasts more tolerant to salinity stress than MC chloroplasts (Omoto et al., 2016). PA production catalyzed by phospholipase D (PLD) under salinity condition is required to phosphorylate MPK6 and subsequent activation of PM Na^+^/H^+^ antiporter (SOS1) and Na^+^ extrusion in *Arabidopsis* (Yu et al., 2010). A previous study reported that the reduced expression of *AtACER*, which encodes ceramidase that hydrolyzes ceramide into sphingosine and FA, disturbs sphingolipid homeostasis and PLD/PA signaling pathway, and thus increases the sensitivity to the salinity stress in *Arabidopsis* (Wu et al., 2015). These results suggested that lipid remodeling plays important roles in the regulation of salt tolerance in plants. Interestingly, a recent study showed that soybean seeds coated with MT enhance salinity tolerance in seedlings possibly by upregulating genes involved in FA biosynthesis (Wei et al., 2015). In addition, a proteomic study revealed that the abundance of several proteins involved in the lipid metabolism pathway was altered by MT in geminating cucumber seeds under salinity conditions (Zhang et al., 2017). These observations implied that MT may alter lipid metabolism in plants under high salinity conditions. However, how MT-remodeled lipid metabolism influences plant salt tolerance is largely unknown.

Given that remodeling of K^+^/Na^+^ homeostasis and lipid metabolism are two important processes involved in the MT improvement of plant salt tolerance, we postulate that MT-remodeled lipid metabolism may be correlated with K^+^/Na^+^ homeostasis regulation in salinized plants. To further elucidate the mechanism through which MT functions in plants exposed to salinity stress, we investigated the effects of exogenous MT on K^+^/Na^+^ homeostasis and lipid metabolism in salinized sweet potato [*Ipomoea batatas* (L.) Lam.], which is an important food and industry crop worldwide, through a combination of multiple analytical techniques. Based on the results presented here, we propose a novel mechanism for MT-mediated salt tolerance. This mechanism highlights the function of MT in stimulating TAG breakdown, FA β-oxidation, and energy turnover under salinity conditions. Thus, MT contributes in improving the energy state and maintain PM H^+^–ATPase activity and K^+^/Na^+^ homeostasis in sweet potato.

## Materials and Methods

### Plant Materials and Treatments

Sweet potato cultivar Xushu 32 (Xu 32, relative salt sensitive) was used in this study, and seedlings were cultured as described previously (Yu et al., 2016). Uniform seedlings with seven to nine mature leaves and tender roots, with length of 8–10 cm, were used for experiments. For salt treatment, the culture solution was added with a required amount of NaCl (final concentration of 150 mM) for 15 days. For exogenous MT treatment, nine different combinations of MT solutions were simultaneously applied to Xu 32 roots (0.1, 0.5, and 1 μM) and leaves (leaf spraying: 50, 100, and 200 μM) 3 days before the NaCl treatment. During the experiment (15 days), root and leaf samples were collected at indicated time points and from the selected MT treatment groups for various measurements. Positive control was provided with the same MT combinations in the absence of NaCl. For pharmacological experiments, diphenyl methylphosphonate (DMP), an inhibitor of the FA β-oxidation pathway (Brown et al., 2013; McLachlan et al., 2016), was added to the roots and leaves during the NaCl treatment.

### Determination of K, Ca, Na, and Mg Contents

The plants were thoroughly washed with deionized water and divided into three parts (the root, stem, and leaf). The fresh samples were dried in an oven at 70°C to constant weight. The dried samples were weighed and pulverized. The pulverized samples were digested with concentrated H_2_O_2_ and HClO_4_ (v:v = 7:1) in a microwave oven (Mars CEM 240/50) and subjected to inductively coupled plasma mass spectrometry analysis (Agilent7500a, United States) to determine the concentrations of K, Ca, Na, and Mg.

### Measurement of K^+^, H^+^, and Na^+^ Fluxes

Net fluxes (K^+^, H^+^, and Na^+^) were determined non-invasively using a non-invasive microtest technique system (NMT-100-SIM-YG, YoungerUSA LLC, Amherst, MA, United States) as described in previous studies (Sun et al., 2009a,b; Yu et al., 2016). The construction of K^+^, H^+^, and Na^+^-selective electrodes followed standard procedures (Sun et al., 2009a,b; Yu et al., 2016). The ion-selective electrodes for the target ions were calibrated before flux measurements: (1) K^+^: 0.1, 0.5, and 1 mM; (2) H^+^: pH 5.0, 6.0, and 7.0; (3) Na^+^: 0.1, 0.5, and 1.0 mM. Electrodes with Nernstian slopes > 50 mV/decade were used. Ion flux was calculated as described previously (Sun et al., 2009a,b; Yu et al., 2016).

#### Transient Ion Flux Measurements

Root tips and mesophyll tissues were collected from MT-pretreated and non-pretreated Xu 32, immobilized, and equilibrated in H^+^ and K^+^ measurement solution (containing 0.1 mM NaCl, 0.1 mM MgCl_2_, 0.1 mM CaCl_2_, and 0.5 mM KCl at pH 5.7) for 30 min. The steady fluxes of H^+^ and K^+^ were recorded for 5 min before salt shock. Afterward, salt shock (150 mM NaCl) was activated by adding NaCl stock (300 mM, pH 5.7, prepared with measurement solution). Transient ion fluxes were monitored for another 30 min in the root apex region (500 μm from the root tip), root mature region (15 mm from the root tip), and mesophyll tissues.

#### Steady-State Ion Flux Measurements

Root segments with a length of ca. 3 cm were sampled from various treatment groups after 1, 3, and 5 days of treatment. The roots were then transferred to the measuring chamber containing 10 mL of fresh measurement solution. The roots were immobilized at the bottom. Na^+^, H^+^, and K^+^ fluxes were monitored in the following measurement solutions (Sun et al., 2009a,b; Yu et al., 2016):

H^+^: 150 mM NaCl, 0.1 mM MgCl_2_, 0.1 mM CaCl_2_, and 0.5 mM KCl, pH 5.7,
K^+^: 150 mM NaCl, 0.1 mM MgCl_2_, 0.1 mM CaCl_2_, and 0.5 mM KCl, pH 5.7,
Na^+^: 0.1 mM NaCl, 0.1 mM MgCl_2_, 0.1 mM CaCl_2_, and 0.5 mM KCl, pH 5.7.

For Na^+^ flux recording, the roots were rinsed with the measurement solution and immediately incubated in Na^+^ measurement solutions to equilibrate for 30 min to decrease the effect of Na^+^ released from the surface of salt-stressed roots. For K^+^ and H^+^ flux recording, 150 mM NaCl was added to the measurement solution to mimic a saline environment (Sun et al., 2009a,b; Yu et al., 2016). In the control roots, 150 mM NaCl in the measurement solution was replaced with 0.1 mM NaCl. Ion fluxes were determined along the root axis in two regions: apex (500–3000 μm from the tip with a measurement interval of 500 μm) and mature zone (10–15 mm from the tip with a measurement interval of 1000 μm). Continuous recording was performed for 2–3 min at each measuring point in the mature and apical regions. Steady-state ion fluxes were expressed as the mean of six measuring points in each region.

### PM Vesicle Purification and PM H^+^–ATPase Activity Determination

Adequate root and leaf samples were collected and homogenized in 20 mL of homogenization buffer containing 250 mM sucrose, 10% (w/v) glycerol, 0.5% (w/v) PVP, 3 mM EDTA, 1 mM DTT, 1 mM PMSF, 15 mM ME, and 25 mM Tris/MES (pH 7.6). The homogenate was filtered through two layers of cotton gauze and centrifuged at 13,000 × *g* for 20 min. The supernatant was re-centrifuged at 80,000 × *g* for 30 min to obtain microsomal membranes. The membranes were re-suspended in buffer containing 1 mM DTT, 1 mM PMSF, and 5 mM Tris/MES (pH 6.5). The microsomal membranes were used to assess ACMA quenching and analyze H^+^ pumping activity (Shabala et al., 2016). The membranes were also used to analyze ATP hydrolysis activity by measuring the release of Pi through the method described by Liu et al. (2014).

### Lipid Droplet Imaging

Mature leaves and tender roots were collected from different treatment groups for liquid droplet (LD) staining. Root tips with 1 cm length and leaf disk with 1 cm diameter were incubated in staining buffer containing 5 mM KCl/MES and 20 μM BODIPY 493/503 (Life Technologies, Carlsbad, CA, United States; D3922) for 20 min. The buffer solution can be used as a stain for neutral lipids and as a tracer for oil body in plants (Cai et al., 2016). The samples were then washed in KCl/MES buffer for 5 min before imaging with an Olympus BX63 epifluorescence microscope.

### Lipase Activity Measurement

Approximately 1 g of fresh leaf samples was ground gently using a mortar and pestle. The homogenate was centrifuged at 100 × *g* for 5 min to pelletize the cell debris. A 200 μL sample of the supernatant (crude extract) was kept for lipase activity assay by using the method described by Eastmond (2006). Protein content was determined using bovine serum albumin as standard (Bradford, 1976). Assays were performed on the supernatant by using an emulsified substrate trieicosenoin, which exhibits high specificity for SDP1 lipase (Eastmond, 2006). The released free FAs were measured using non-esterified fatty acid (NEFA) colorimetric kit according to the manufacturer’s instructions.

### Lipid Extraction and Lipidomic Analysis

Fresh leaf samples (0.1 g) were ground in liquid nitrogen to fine powder and extracted by 1.4 mL of 100% isopropanol. The mixture was transferred into 2 mL centrifuge tubes for vortex oscillation for 10 s and ultrasonic treatment for 10 min. The mixture was frozen at -20°C for 1 h and oscillated at room temperature. The samples were then centrifuged at 10,000 × *g* at 4°C for 20 min. The supernatant was filtered by a 0.22 μM filter and transferred to a glass vial for UPLC-ESI-QTOF-MS analysis. Each sample (2 μL) was injected into a reverse-phase CSH C18 column (1.7 μm, 1 mm × 50 mm) by using an Acquity *I*-class UPLC system (Waters Corporation, United States). The column oven temperature was set at 55°C. The mobile phase comprised acetonitrile (ACN)/H_2_O (60%/40%) containing 0.1% formic acid and 10 mM ammonium formate (solvent A) and IPA/ACN containing 0.1% formic acid and 10 mM ammonium formate (90%/10%) (solvent B). Each sample was resolved for 20 min at a flow rate of 0.4 mL/min. The UPLC gradient started with 40% B and then ramped to 43% B from 0 to 2 min, followed by ramp up to 50% B from 2 to 2.1 min, 54% B from 2.1 to 12 min, 70% B from 12 to 12.1 min, 99% B from 12.1 to 18 min, and finally a ramp to initial 40% B from 18 to 18.1 min held for 2 min.

Mass spectrometry was performed on a Q-TOF instrument (Xevo G2-S QTOF, Waters Corporation, United States) operated in either negative (ESI-) or positive (ESI+) electrospray ionization mode with a capillary voltage of 3 kV and a sampling cone voltage of 25 V in both modes. The desolvation gas flow was set to 800 L/h, and the temperature was set to 500°C. The source temperature was set to 120°C. Accurate mass was maintained by introducing a lock-spray interface of leucine–enkephalin (556.2771 [M+H]+ or 554.2615 [M-H]-). Data were acquired in continuum multistep excitation mode from 50 m/z to 1500 m/z mass range. The pooled quality control (QC) samples (generated by taking an equal aliquot of all samples included in the experiment) were run at the beginning of the sample queue for column conditioning and every 10 injections thereafter as described in the study of Mapstone et al. (2014). A test mix of standard metabolites was run at the beginning and at the end of the process to evaluate instrument performance with respect to sensitivity and mass accuracy (Mapstone et al., 2014). The overlay of the total ion chromatograms of the QC samples showed excellent retention time reproducibility. The sample queue was randomized to remove bias. The m/z features of metabolites were normalized with log transformation that stabilized the variance, followed by quantile normalization to ensure uniform empirical distribution of intensities across the samples. The metabolites were selected among all those identifiable by using receiver operating characteristic-regularized learning technique based on the LASSO penalty as implemented with R package “glmnet,” which uses a cyclical coordinate descent in a path-wise manner (Mapstone et al., 2014).

### RNA Extraction and Quantitative Real-Time Polymerase Chain Reaction (PCR)

Total RNA was isolated from the root and leaf tissues by using Trizol reagent (Takara Bio Inc., Japan). Quantitative real-time PCR was performed as described previously (Yu et al., 2016). The PCR products were sequenced and validated. Primers designed to target genes (Supplementary Table S1), including *DGAT1* (acylCoA:diacylglycerol acyltransferase), *PDAT1* (phospholipid:diacylglycerol acyltransferase), *LACS6-7* (long-chain acyl-CoA synthetases), *ACX1-4* (acyl-coA oxidase), *CSY2* (citrate synthase), *MLS* (malate synthase), *ICL* (isocitrate lyase), and *SDP1* (TAG lipase), were established based on the transcriptome sequences of sweet potato. The relative expression level of each target gene was normalized to that of *IbUBI* (GenBank Accession Number: JX177358.1), a stable internal reference gene of *I. batatas*. This gene was amplified using the following primers: forward primer 5′-TCGACAATGTGAAGGCAAAG-3′ and reverse primer 5′-CTTGATCTTCTTCGGCTTGG-3′. Relative expression levels were calculated using 2^-ΔΔCt^ method.

### ATP Content Measurement

The root and leaf tissues were ground to fine powder in liquid nitrogen. Subsequently, 50 mg of the root and leaf tissues were homogenized with 500 μL of 0.1 M HCl for 5 min. The homogenate was centrifuged at 18,000 × *g* for 10 min, and the supernatant was centrifuged again at 14,000 × *g* for 20 min. ATP content was determined using an ATP assay kit (Promega). Relative ATP level was expressed as normalized luminescence.

### Statistical Analysis

Data were subjected to ANOVA. Significant differences between means were determined by Duncan’s multiple range test. Unless otherwise stated, differences at *P* < 0.05 were considered significant.

## Results

### Exogenous MT Enhanced Salt Resistance in Sweet Potato Seedlings

In our preliminary experiments, different combinations of exogenous MT decreased the NaCl-elevated electrolyte leakage, malondialdehyde content, superoxide anion production rate, hydrogen peroxide and proline content in Xu 32 leaves (Supplementary Figure S1). In addition, MT enhanced the chlorophyll content, relative water content, and the antioxidant enzyme activities (catalase and superoxide dismutase) in Xu 32 leaves after 7 days of salinity stress (Supplementary Figure S1). These results indicated that salt tolerance in Xu 32 seedlings was improved by exogenous MT. The beneficial effects of MT became apparent with the application of 0.5/100 μM (root/leaf) or 1.0/100 μM MT under 150 mM NaCl condition. Therefore, the two MT combinations were used for further experiments.

### Effects of MT on K^+^, Ca^2+^, Na^+^, and Mg^2+^ Contents

**FIGURE 1:**
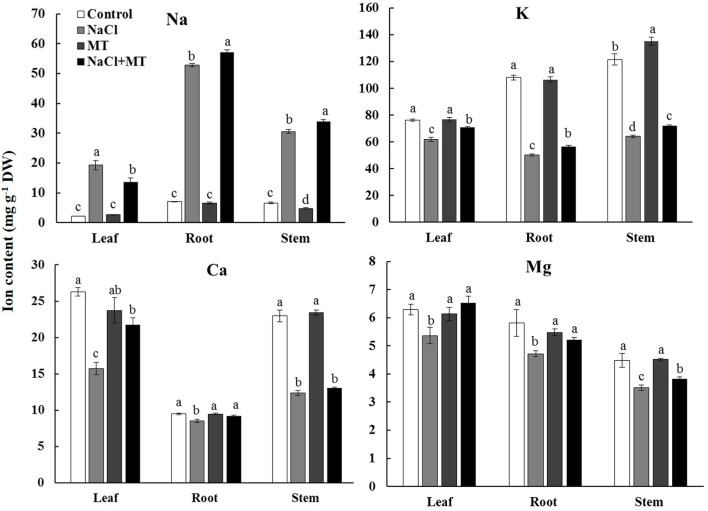
Effects of MT on the K^+^, Na^+^, Ca^2+^, and Mg^2+^ contents in NaCl-stressed or non-stressed Xu 32. Xu 32 seedlings were pretreated with 0.5 μM (root) + 100 μM (leaf) MT for 3 days before being subjected to the 150 mM NaCl stress. K^+^, Na^+^, Ca^2+^, and Mg^2+^ contents were analyzed after 7 days of salinity treatment. Columns represent the means of independent measurements of five individual plants per treatment, and bars represent the standard error of the mean. Columns labeled with different letters indicate significant difference at *P* < 0.05.

NaCl treatment for 7 days significantly decreased the contents of K^+^, Ca^2+^, and Mg^2+^ but increased the Na^+^ content in the root, stem, and leaf tissues of Xu 32 (**Figure 1**). Exogenous application of MT (root/leaf: 0.5/100 μM) partially reversed this depletive effect of salt on the K^+^, Ca^2+^, and Mg^2+^ contents in different tissues (**Figure 1**). Interestingly, MT markedly increased the Na^+^ content in the root and stem tissues but significantly decreased the Na^+^ content in the salinized leaf tissues of Xu 32 (**Figure 1**). MT did not alter Na^+^, K^+^, Ca^2+^, and Mg^2+^ levels in the absence of NaCl stress (**Figure 1**).

### Effects of MT on K^+^ and H^+^ Fluxes in the Root and Mesophyll Tissues

**FIGURE 2:**
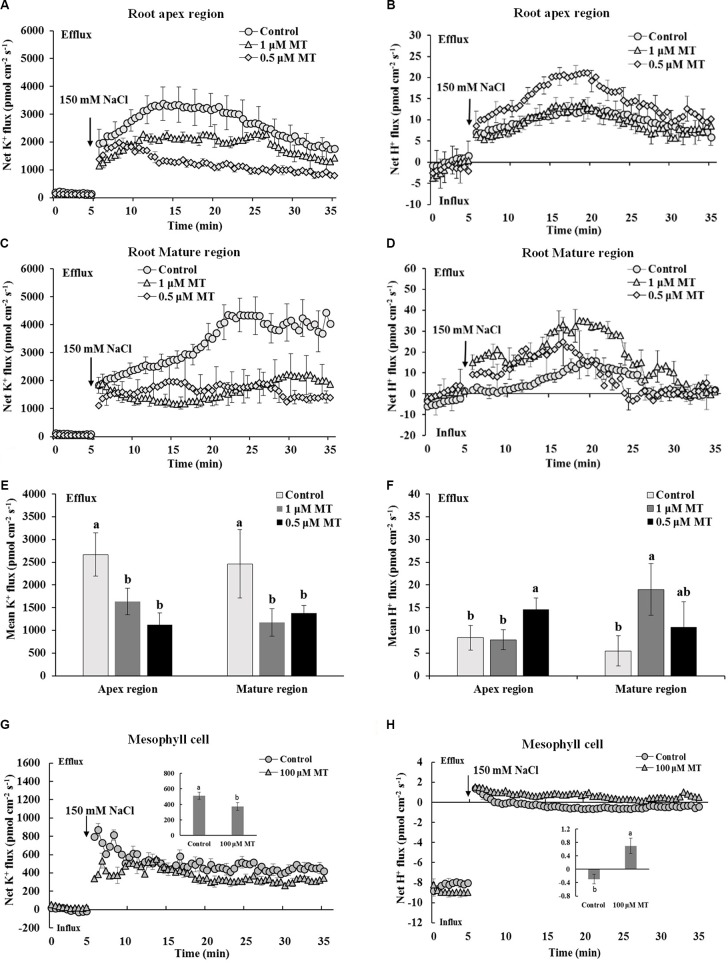
Effects of MT pretreatment on the salt shock-triggered transient K^+^ and H^+^ flux in the root and mesophyll tissue of Xu 32. **(A,C)** Transient K^+^ flux upon 150 mM NaCl shock in the root apex region (measured at 500 μm from the tip) and mature region (measured at 15 mm from the tip) of MT-pretreated (0.5 and 1.0 μM) or non-pretreated seedlings. **(B,D)** Transient H^+^ flux. **(A–D)** Each point represents the mean of eight roots at minimum collected from four individual plants. **(E,F)** Columns show the mean K^+^ and H^+^ flux during the period of NaCl shock (**A–D**, approximately 30 min). Different letters denote significant differences at *P* < 0.05. **(G,H)** Transient kinetics of K^+^ and H^+^ flux upon 150 mM NaCl shock in mesophyll tissue of MT-pretreated (100 μM) or non-pretreated seedlings. Each point represents the mean of a minimum of eight replicates. Inserted columns show the mean K^+^ and H^+^ flux during the period of NaCl shock (approximately 30 min). Different letters denote significant differences at *P* < 0.05.

Transient K^+^ and H^+^ flux responses were measured in the root and mesophyll tissues to determine the possible mechanisms involved in MT-improved K^+^/Na^+^ homeostasis in Xu 32. NaCl shock triggered a strong net K^+^ efflux in the root apex region, mature region, and mesophyll tissues. MT pretreatment (root/leaf: 0.5/100 μM or 1.0/100 μM) for 72 h significantly decreased the NaCl-induced transient K^+^ efflux by 35–60% in different tissues (**Figures 2A,C,E,G**). NaCl shock increased the net H^+^ efflux in the different regions of the root (**Figures 2B,D,F**). MT pretreatment increased the NaCl-induced net H^+^ efflux by 75% in the root apex region and by 375% in the root mature region (**Figures 2B,D,F**). Similarly, the MT-stimulated H^+^ efflux upon salt shock was observed in mesophyll tissues (**Figure 2H**). The MT-altered K^+^/H^+^ flux upon NaCl shock was reversed in the presence of vanadate (Supplementary Figure S2), which is an inhibitor of PM H^+^–ATPase. Thus, MT mediated K^+^/H^+^ flux by upregulating the PM H^+^–ATPase activity (Supplementary Figure S2).

**FIGURE 3:**
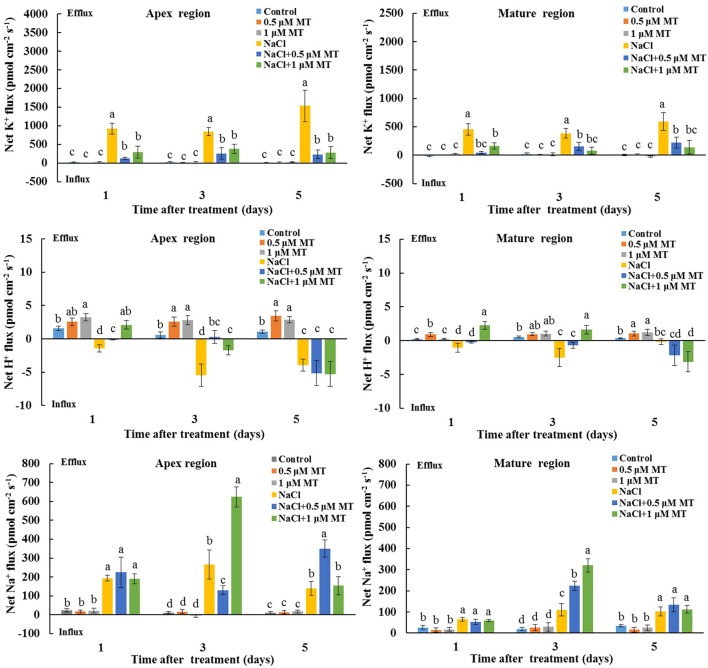
Effects of MT on steady-state fluxes of K^+^, H^+^, and Na^+^ in root different regions of NaCl-stressed or non-stressed Xu 32. Xu 32 seedlings were pretreated with 0.5 μM (root) + 100 μM (leaf) MT or 1.0 μM (root) +100 μM (leaf) MT for 3 days and then subjected to the 150 mM NaCl stress. The steady-state fluxes at the root apex (500–3000 μm from the tip) and mature region (10–15 mm from the tip) were measured after 1, 3, and 5 days of NaCl treatment. Each column is the mean of a minimum of 10 roots collected from five individual seedlings, and bars represent the standard errors of the mean. Columns labeled with different letters indicate significant difference at *P* < 0.05.

We also tested the K^+^/H^+^/Na^+^ flux responses under prolonged salinity conditions. A steady K^+^ efflux was observed in the root apex and mature regions after 1, 3, and 5 days of NaCl treatment (**Figure 3**). This NaCl-triggered sustaining K^+^ efflux was significantly inhibited in the presence of 0.5 and 1 μM MT (**Figure 3**). We also observed that MT enhanced steady-state H^+^ efflux in salinized Xu 32 roots at the first day of NaCl stress (**Figure 3**). In addition, the rate of net Na^+^ efflux in salinized Xu 32 roots was significantly enhanced in the presence of MT (**Figure 3**).

### Effects of MT on the PM H^+^–ATPase Activity and ATP Level

**FIGURE 4:**
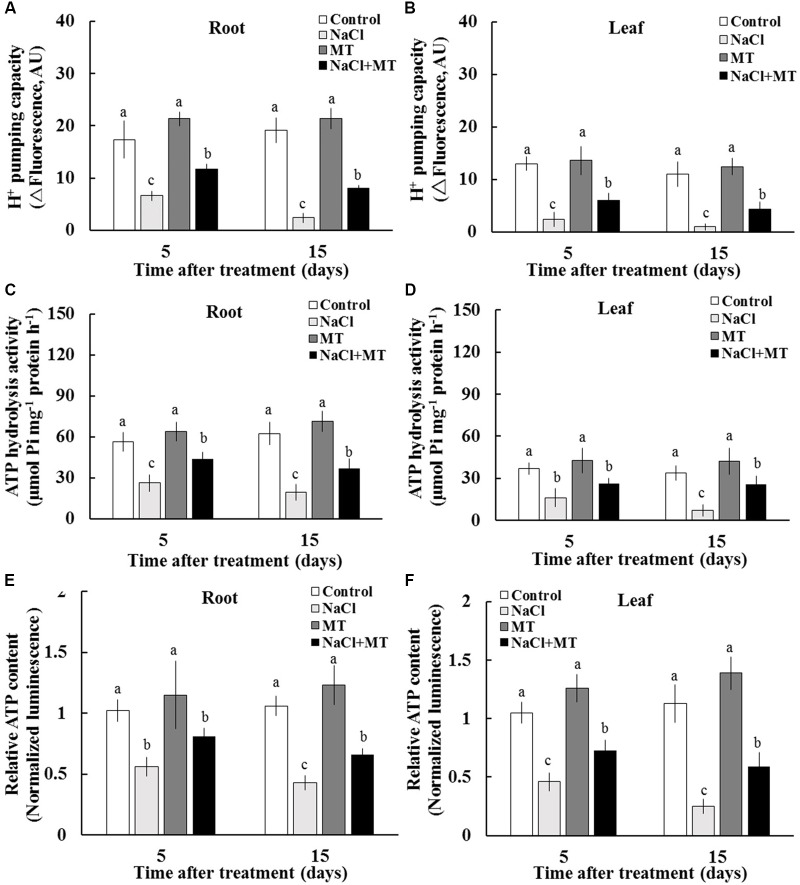
Effects of MT on the PM H^+^–ATPase activity and ATP level in the root and leaf tissues of NaCl-stressed or non-stressed Xu 32. Xu 32 seedlings were pretreated with 0.5 μM (root) + 100 μM (leaf) MT for 3 days and then subjected to the 150 mM NaCl stress. **(A,B)** H^+^ pumping activity in PM vesicles purified from the root and leaf tissues of Xu 32 after 5 and 15 days of NaCl treatment. **(C,D)** ATP hydrolysis activity. **(A–D)** Each column is the mean of three independent experiments. **(E,F)** Relative ATP level. Each column is the mean of six individual plants. Columns labeled with different letters indicate significant difference at *P* < 0.05.

We purified PM vesicles from the root and leaf tissues collected from different treatments, followed by an H^+^–ATPase activity assay by using ACMA-based method and Pi releasing-based method. PM vesicles purified from control and MT-treated plants exhibited high PM H^+^ pumping capacity, as indicated by the decreasing fluorescence of ACMA after H^+^–ATPase activation by Mg^2+^ (**Figures 4A,B**). However, PM vesicles purified from salinized root and leaf tissue exhibited a significant inhibition of Mg^2+^-induced fluorescence quenching (**Figures 4A,B**), indicating the markedly decreased PM H^+^ pumping capacity. The PM H^+^ pumping capacity was significantly enhanced by MT in salinized tissues (**Figures 4A,B**). Salinity treatment decreased the ATP hydrolysis activity of PM H^+^–ATPase in both the root and leaf tissues. However, the ATP hydrolysis activity was markedly high in the presence of MT under salinity condition (**Figures 4C,D**). In addition, salinity stress markedly reduced the ATP content in sweet potato vegetative tissues, whereas the ATP level was markedly higher in the NaCl+MT-treated plants than that in the NaCl-treated plants (**Figures 4E,F**).

### Effects of MT on the Leaf Lipidome

**FIGURE 5:**
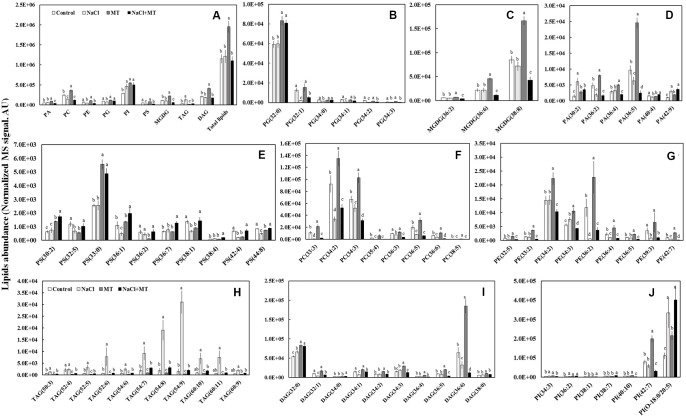
Effects of MT on the leaf lipidome in NaCl-stressed or non-stressed Xu 32. Xu 32 seedlings were pretreated with 0.5 μM (root) + 100 μM (leaf) MT for 3 days and then subjected to 150 mM NaCl stress. The mature leaves were collected at the same position after 7 days of NaCl treatment for lipidomic profiling. **(A)** Total amount of lipids in various head group classes. **(B–J)** Abundance of lipid molecular species of nine main lipid classes. Each column is the mean of five repetitions, and columns labeled with different letters indicate significant difference at *P* < 0.05.

We further profiled the molecular species of total lipids extracted from control-, NaCl-, MT-, and NaCl+MT-treated Xu 32 leaves (7 days after treatment) by using a lipidomic approach based on UPLC/ESI-QTOF/MS. We found nine main classes of lipids that contained 70 molecular species, the abundance of which showed significant difference when compared between any two treatments. The nine main classes included six classes of phospholipids (PA; phosphatidylglycerol, PG; phosphatidylserine, PS; phosphatidylcholine, PC; phosphatidylinositol, PI; and phosphatidyl ethanolamine, PE), one class of galactolipids (MGDG), and two classes of glyceride (TAG and DAG) (**Figure 5**).

The level of total lipids increased significantly in MT-treated leaves when compared with control (**Figure 5A**). The MT-increased leaf total lipids is due largely to the elevation of PC (mainly 34:2, 34:3, and 36:5 species), PI (mainly 42:7 species), MGDG (mainly 36:6 and 38:8 species), and DAG (mainly 32:0 and 36:6 species) (**Figures 5A,C,F,I,J**). However, MT significantly decreased the abundance of TAG (**Figures 5A,H**). Salinity treatment did not influence the level of total lipids but significantly decreased the abundance of PC, PE, PG, and PS (**Figure 5A**). This salt-induced reduction of phospholipids coincided with an increase in the relative abundance of TAG (mainly 52:6, 54:7, 54:8, 54:9, 60:10 and 60:11 species) (**Figures 5A,H**). Interestingly, the salt-increased TAG accumulation in Xu 32 leaves was almost completely abolished by MT (**Figures 5A,H**).

### Effects of MT on the Expression Levels of Genes Related to the TAG Mobilization

**FIGURE 6:**
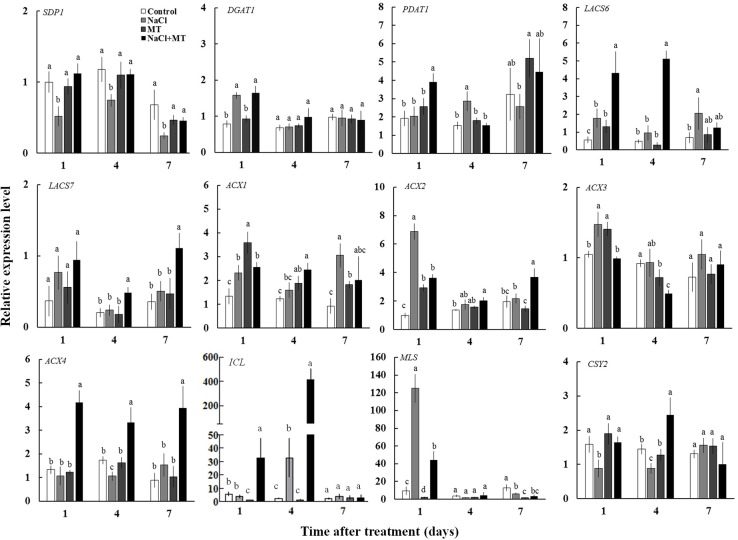
Effects of MT on the expression level of genes related to the TAG synthesis and breakdown, FA β-oxidation, and energy turnover in the leaves of NaCl-stressed or non-stressed Xu 32. Xu 32 seedlings were pretreated with 0.5 μM (root) + 100 μM (leaf) MT for 3 days and then subjected to 150 mM NaCl stress. The mature leaves were collected at the same position after 1, 4, and 7 days of NaCl treatment. Each column is the mean of four replications, and columns labeled with different letters indicate significant difference at *P* < 0.05.

To explore the reason of inhibitory effect of MT on the TAG accumulation under salinity condition, we examined the expression levels of genes related to TAG synthesis (*DGAT1* and *PDAT1*) and breakdown (*SDP1*) in Xu 32 leaves. Salinity stress significantly enhanced the expression level of *DGAT1* and *PDAT1* (**Figure 6**). Compared with the NaCl treatment group, MT did not influence the expression of *DGAT1* but enhanced the mRNA abundance of *PDAT1* on the first day of NaCl stress (**Figure 6**). The expression level of *SDP1*, which encodes the main TAG lipase in plants (Eastmond, 2006), was significantly downregulated under salinity condition (**Figure 6**). MT recovered the expression of *SDP1* to the control level under high salinity (**Figure 6**). Similarly, MT reversed the inhibitory effect of high salinity on the lipase activity in Xu 32 leaves (Supplementary Figure S3). MT did not influence the expression of these three genes in the absence of NaCl stress (**Figure 6**).

In addition, the mRNA abundance of *LACS6*, *LACS7*, *ACX4*, *ICL*, and *CSY2*, which are important genes involved in FA β-oxidation and energy turnover in plants, were significantly higher in the leaves of NaCl plus MT-treated seedlings than that in NaCl-treated seedlings (**Figure 6**). Interestingly, NaCl stress markedly enhanced *MLS* expression level at the first day of treatment when compared with control seedlings. This salt-induced high expression of *MLS* was significantly inhibited by MT (**Figure 6**).

### Inhibition of TAG Mobilization under Salinity Stress Disrupted K^+^/Na^+^ Homeostasis and PM H^+^–ATPase Activity

**FIGURE 7:**
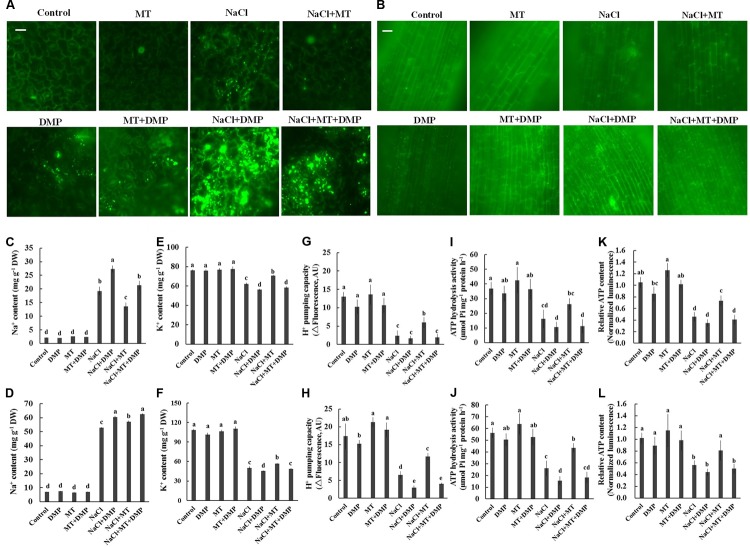
DMP reversed the effects of MT on the TAG accumulation, K^+^ and Na^+^ content, PM H^+^–ATPase activity, and cellular ATP level in NaCl-stressed Xu 32. Xu 32 seedlings were pretreated with 0.5 μM (root) + 100 μM (leaf) MT for 3 days and then subjected to NaCl (150 mM) plus DMP (25 μM) treatment. **(A,B)** Mature leaves and fine roots were collected from various treatment groups (5 days after treatment), stained with BODIPY 493/503 and then observed under a fluorescence microscope. For each treatment, a minimum of 10 root segments and leaf disks from four individual plants was observed. Representative images showing the alteration of lipid droplet accumulation. **(C–F)** Na^+^ and K^+^ content in the leaves **(C,E)** and roots **(D,F)** of Xu 32 under various treatment conditions (7 days after treatment). Columns represent the means of five repetitions, whereas bars represent the standard error of the mean. **(G–J)** H^+^ pumping and ATP hydrolysis activity in PM vesicles purified from the leaves **(G,I)** and roots **(H,J)** of Xu 32 under different conditions (5 days after treatment). Each column is the mean of three independent experiments. **(K,L)** Relative ATP level in the leaves **(K)** and roots **(L)** of Xu 32 under different conditions (5 days after treatment). Each column is the mean of six repetitions. Columns labeled with different letters indicate significant difference at *P* < 0.05.

Diphenyl methylphosphonate, a specific inhibitor of TAG mobilization in plants (Brown et al., 2013; McLachlan et al., 2016), was selected to confirm the role of TAG metabolism in MT-improved K^+^/Na^+^ homeostasis in sweet potato. BODIPY and fluorescence microscopy were adopted to indicate LD accumulation. No LD-specific fluorescence was observed in the control and MT-treated leaf and root cells. However, a large increase in the number and volume of BODIPY-stained LDs was observed after NaCl stress (**Figures 7A,B**). This salt-increased LDs largely decreased in the presence of MT (**Figures 7A,B**), indicating the reduction of TAG accumulation. However, the BODIPY-stained LDs were significantly enhanced in the presence of DMP (**Figures 7A,B**). Meanwhile, DMP treatment significantly enhanced Na^+^ accumulation and decreased K^+^ level in the root and leaf tissues of NaCl- and NaCl+ MT-treated seedlings (**Figures 7C–F**). The MT-dependent recovery of H^+^ pumping capacity, ATP hydrolysis activity, and relative ATP level in the root and leaf tissues of salinized Xu 32 also significantly reduced in the presence of DMP (**Figures 7G–L**). DMP did not alter ion content and PM H^+^–ATPase activity in the absence of NaCl treatment (**Figures 7G–L**).

## Discussion

Melatonin is a multifunction compound proposed to be an important regulator in controlling plant stress responses (Zhang et al., 2015). Recently, the beneficial roles of MT on plant salinity tolerance has been gradually revealed mainly through two methods, namely, exogenous application of MT or genetic modification of enzymes involved in MT synthesis (Li et al., 2012, 2016; Liang et al., 2015; Shi et al., 2015; Wei et al., 2015; Byeon and Back, 2016; Chen et al., 2017; Zhang et al., 2017). In this work, by using exogenous application method, we revealed a novel role of MT in linking lipid metabolism with K^+^/Na^+^ homeostasis regulation in salinized sweet potato.

Increasing evidence shows that a high capacity for maintaining cytosolic K^+^ homeostasis is an important salt-resistant trait in plants (Chen et al., 2005; Cuin et al., 2008; Sun et al., 2009b; Anschütz et al., 2014; Shabala and Pottosin, 2014). In our previous report, a weak capacity for K^+^ retention was observed in Xu 32 compared with a salt-tolerant sweet potato cultivar (Yu et al., 2016). Here, we showed that the ability for K^+^ retention in salinized Xu 32 was markedly enhanced by exogenous MT (**Figure 1**). This result was consistent with previous observation in *Malus* plants (Li et al., 2016). The maintenance of high cytosolic K^+^ levels is essential to maintain appropriate metabolic processes and the activity of membrane transporters, such as vacuolar proton pumps, thus enabling the establishment of proton motive force across the tonoplast and facilitating vacuolar Na^+^ and Cl^-^ sequestration under salinity condition (Shabala, 2013). Correspondingly, we found that MT enhanced the Na^+^ accumulation in roots of salinized Xu 32 (**Figure 1**). This phenomenon is probably ascribed to the MT-enhanced Na^+^/H^+^ antiport activity across the tonoplast in roots (Li et al., 2012). Interestingly, we observed that MT reduced Na^+^ accumulation in leaves but enhanced Na^+^ content in stems of salinized Xu 32 (**Figure 1**). This is probably ascribed to the MT-restricted root-to-shoot translocation of Na^+^. However, the underlying mechanisms still need further investigation. K^+^ also is a major inorganic osmolyte that confers the majority of tissue osmotic adjustment under stress conditions (Shabala and Lew, 2002). In *Malus* plants, the MT-improved K^+^ homeostasis is ascribed to the enhanced K^+^ uptake and is achieved mainly by CBL1–CIPK23 pathway-mediated upregulation of K^+^ transporter genes, such as *AKT1* and *HKT1* (Li et al., 2016). The results presented here showed that exogenous MT performs an important role in reducing NaCl-triggered K^+^ efflux in sweet potato vegetative tissues (**Figures 2**, **3**). This action of MT may serve as an important mechanism in the maintenance of plant K^+^ homeostasis under salinity condition.

The extent of salt-triggered K^+^ efflux in plants was largely dependent on two factors, namely, PM H^+^–ATPase activity and ROS accumulation (Shabala, 2013; Anschütz et al., 2014; Shabala and Pottosin, 2014). Intrinsically, high H^+^–ATPase activity is essential to prevent the depolarization of membrane potential, which activates voltage-dependent outward K^+^ channels and K^+^ efflux under high external Na^+^ (Chen et al., 2007; Sun et al., 2009b; Shabala, 2013; Anschütz et al., 2014; Shabala and Pottosin, 2014; Yu et al., 2016). In this study, several lines of evidence proved that the MT reduction of K^+^ efflux in salinized Xu 32 is mediated by the enhanced PM H^+^–ATPase activity. First, compared with non-MT-treated Xu 32, MT pretreatment resulted in an evidently enhanced H^+^ efflux that coincided with a decreased K^+^ efflux upon NaCl shock (**Figure 2**). This pattern of K^+^/H^+^ flux under NaCl shock has been ascribed to enhanced PM H^+^–ATPase activity (Sun et al., 2009b; Yu et al., 2016; Azhar et al., 2017). Second, the PM H^+^–ATPase inhibitor enlarged the salt shock-induced K^+^ efflux in MT-treated Xu 32 root and MCs (Supplementary Figure S2). Finally, direct measurement of H^+^ pumping and ATP hydrolysis capacity proved that MT reversed the inhibition effect of salinity on the activity of PM H^+^–ATPase (**Figure 4**). Therefore, the increased PM H^+^–ATPase activity triggered by MT may offer less depolarized membrane potential and less activated outward K^+^ channels, thereby leading to a less reduction of cytosolic K^+^ in salinized Xu 32.

Melatonin exhibits strong anti-oxidative ability to reduce oxidative damage caused by various environment stresses, including salinity (Liang et al., 2015; Han et al., 2017; Li et al., 2017; Zheng et al., 2017). In this study, the antioxidant enzyme activities were enhanced by MT, whereas ROS accumulation was inhibited by MT in salinized Xu 32 leaves (Supplementary Figure S1). This finding may contribute to the protection of photosynthetic machinery from salinity-triggered oxidative damage in Xu 32 (Rangani et al., 2016). ROS, such as H_2_O_2_ and hydroxyl radicals, which are triggered by high salinity, usually cause a major perturbation in intracellular K^+^ homeostasis by activating a range of K^+^-permeable channels (Shabala and Pottosin, 2014). Thus, the good capacity of K^+^ maintenance in MT-primed Xu 32 should be partially ascribed to the MT-enhanced ROS scavenging capacity and subsequent deactivation of ROS-dependent K^+^ permeable channels.

Lipid remodeling refers to decreases in the amounts of certain lipids and increases in others. This process performs an important role in the adaptation of plants to environmental stress (Tenenboim et al., 2016). Various abiotic stresses can induce TAG accumulation in plant vegetative tissues (Abida et al., 2015; Mueller et al., 2015). A massive accumulation of TAG is frequently observed in senescing leaves of plants under different conditions (Watanabe et al., 2013; Kim et al., 2015). In our unpublished lipidomic data, the salt-increased TAG in Xu 32 leaves was markedly higher than that in a salt-tolerant cultivar (data not shown). Thus, we suggested that salt-triggered TAG accumulation in Xu 32 leaves should be a hallmark of senescence and reflects low salt resistance of the plant. In the present study, MT application almost completely blocked the salt-triggered TAG accumulation in Xu 32 leaves, suggesting the superior physiological state of these seedlings. Here, we observed that the expression level of *SDP1*, which is an oil body-associated TAG lipase with a patatin-like acyl-hydrolase domain and release free FA from oil body in young seedlings and vegetative tissues of adult plants (Eastmond, 2006; Hu et al., 2012; Fan et al., 2014; Xu and Shanklin, 2016), and lipase activity in salinized Xu 32 leaves were all recovered to the control level by MT (**Figure 6**). Thus, these results clearly showed that MT performs a significant role in stimulating TAG breakdown via enhancing *SDP1* gene expression and lipase activity in salinized Xu 32 leaves. This function of MT observed here is consistent with recent studies found in animal oocytes and germinating cucumber seeds (Jin et al., 2017; Zhang et al., 2017).

**FIGURE 8:**
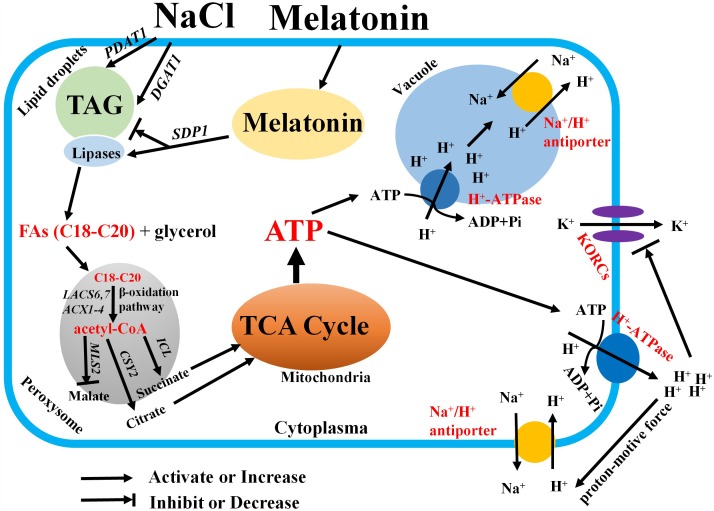
Schematic model showing the mediation of MT on salt tolerance in sweet potato. In this model, MT recovers the inhibitory effects of salt stress on the expression of *SDP1* and lipase activity and accelerates FA release from TAG. Thus, several free FAs with appropriate acyl chain are transported to the peroxisome for β-oxidation. The resulting acetyl-CoA is used for catalyzing the production of succinates and citrates by isocitrate lyase and citrate synthase. Then, succinate and citrate enter the mitochondria for the TCA cycle. Thus, abundant ATPs were generated for driving the activity of H^+^–ATPase across the PM and tonoplast. Therefore, the increased PM H^+^–ATPase activity blocks the K^+^ efflux via depolarization-activated K^+^ outward channels. In addition, the Na^+^/H^+^ antiport activity across the PM and tonoplast is fuelled by the generated proton motive force. Finally, the K^+^/Na^+^ homeostasis is maintained under salinity stress.

Following TAG hydrolysis by lipases, FA is transported and entered into the peroxisome for β-oxidation (Hu et al., 2012; Xu and Shanklin, 2016). In the present study, we show that the expression levels of β-oxidation marker genes (*LACS6*, *LACS7*, and *ACX4*) were markedly enhanced by MT in the presence of salinity stress (**Figure 6**). This result indicated that MT enhances FA β-oxidation pathway in the peroxisomes of salinized Xu 32 leaves (Hu et al., 2012; Xu and Shanklin, 2016). Of note, H_2_O_2_ production associated with acyl-CoA oxidase activity from FA β-oxidation may results in subcellular oxidative stress (Hu et al., 2012). The MT-upregulation of antioxidant enzymes activities may contribute to the elimination of additional H_2_O_2_ and reestablishment of ROS homeostasis (Supplementary Figure S1). Acetyl-CoA, which is generated by the FA β-oxidation pathway, is used for energy production via mitochondrial respiration and for the synthesis of carbon skeletons via the glyoxylate cycle and gluconeogenesis (Hu et al., 2012; Xu and Shanklin, 2016). In this study, *CSY2*, which is the gene encoding citrate synthase in sweet potato, was elevated by MT in salinized Xu 32 leaves (**Figure 6**), suggesting increased citrate production used in the tricarboxylic acid (TCA) cycle and energy production. This finding was consistent with a recent report, wherein β-oxidation product acetyl-CoA is converted to citrate and used in mitochondrial respiration in light-induced stomatal opening (McLachlan et al., 2016). In addition, we found that salinity stress significantly enhanced the expression of genes encoding malate synthetase (*MLS*) and isocitrate lyase (*ICL*), which are two important enzymes involved in glyoxylate cycle (Hu et al., 2012; Xu and Shanklin, 2016) (**Figure 6**). In general, the glyoxylate cycle activity appears to be absent in mature leaves under most conditions (Hu et al., 2012; Xu and Shanklin, 2016). The activities of malate synthase and isocitrate lyase are induced in the leaves of different plant species during senescence (Graham et al., 1992; Landolt and Matile, 1999). Thus, the salinity-induced high expression of these two genes should be a reasonable response that reflects the beginning of leaf senescence. However, the salt-induced expression pattern of these two genes was differentially affected by MT (**Figure 6**). MT downregulation of *MLS* and MT upregulation of *ICL* in salinized Xu 32 suggested that the increased acetyl-CoA produced via FA β-oxidation was used to synthesize succinate, which can enter the mitochondria for TCA cycle and energy production (**Figure 6**). Although we did not measure organic acid content in this study, the action of MT on the expression of *ICL/MLS/CSY2* in salinized Xu 32 leaves is thought to be beneficial to organic acid homeostasis in peroxisomes and subsequent energy turnover in the mitochondria. Consistent with this notion, the energy state in salinized Xu 32 was markedly enhanced in the presence of MT (**Figure 4**). The good energy state is essential for the ATP-dependent defensive responses, including the maintenance of proton pump activity across the PM and tonoplast (Munns and Tester, 2008; Yu et al., 2016). As direct evidence, the MT-enhanced energy state, PM H^+^–ATPase activity, and K^+^/Na^+^ homeostasis in salinized Xu 32 were all impaired when FA β-oxidation pathway was inhibited by DMP (**Figure 7**). These results clearly revealed that MT functions in stimulating TAG breakdown, FA β-oxidation, and energy turnover under salinity conditions. Thus, MT contributed to a good energy state, which is required for the maintenance of PM H^+^–ATPase activity and K^+^/Na^+^ homeostasis (**Figure 8**).

## Conclusion

We provide a novel insight into the mechanism of MT on the regulation of plant salt tolerance (**Figure 8**). Considering the vegetative propagation of cultivated sweet potato, this novel role of MT found in vegetative tissues may mirror a similar function of MT in geminating seeds and young seedlings of other crops. Therefore, MT may help other crops that reproduce via seeds to generate increased energy from the stored oils and to soundly withstand salinity or other hostile conditions during germination.

## Author Contributions

JS and ZL conceived and designed the experiment. YY, AW, and XL carried out the experiment. MK offered the plant material. WW and XC offered the technological support on lipidomics. JS wrote the manuscript. TX, MZ, DM, and ZL helped to revise the manuscript. All authors read and approved the manuscript.

## Conflict of Interest Statement

The authors declare that the research was conducted in the absence of any commercial or financial relationships that could be construed as a potential conflict of interest.

## Abbreviations

DAG: diglyceride
FA: fatty acid
MGDG: monoglycosyldiglyceride
MT: melatonin
NMT: non-invasive microtest technique
PA: phosphatidic acid
PC: phosphatidylcholine
PE: phosphatidylethanolamine
PG: phosphatidylglycerol
PI: phosphatidylinositol
PM: plasma membrane
PS: phosphatidylserine
TAG: triacylglycerol
UPLC/ESI-QTOF/MS: ultra performance liquid chromatography/electrospray ionization-quadrupole time-of-flight/Mass spectrum.
