# Supplementary material for: Melatonin-Stimulated Triacylglycerol Breakdown and Energy Turnover under Salinity Stress Contributes to the Maintenance of Plasma Membrane H+–ATPase Activity and K+/Na+ Homeostasis in Sweet Potato
Source: Front Plant Sci. 2018 Feb 27;9:256. doi: 10.3389/fpls.2018.00256 (PMC5835075; doi:10.3389/fpls.2018.00256)
Supplement: Supplementary file 1 [file Table_1.DOCX]

Table S1 Primers used in this study

| Gene | Forward primers(5'-3') | Reverse primers(5'-3') |
| --- | --- | --- |
| *DGAT1* | TGCCTGTTCATAAGTGGATGGT | ACATAATTCCAATAAATGCCCAGA |
| *PDAT1* | AGTCGATACTGAGGCGGAGAAAGG | CATGAACAACAGCACCCACCAAAT |
| *SDP1* | AGGGCGATGTGACAGTTGTGATG | TCGACGCATGTGGTTGAGTATTG |
| *MLS* | CTTGCGGAAGTTGTTATAGTTGTG | GGTGAATTTGAGGGACGCTGTT |
| *CSY2* | TGAGACCTTTATCATTCTTGCCTGTT | CGAAGCCTCGGTTGTATTAGCG |
| *LACS6* | GGGGATACGACACCGTCTCATT | AGTTTCTCGGGAAGCACAACAC |
| *LACS7* | GTGATCTCGCAGACTCTGGAGC | ATGAGCGGAAGTTTACCCAATA |
| *ICL* | AGTGCCCAGAATGACCCAATAC | GTTCCAGGAATCATGGACAAGG |
| *ACX1* | CTTCATTGGGCGATGTTTATTC | ATCAAATGTGGCAGTGGTTTCT |
| *ACX2* | AAACCGATTAACAGCAGCATAGC | TATCAGTCTCACCAGCACAAGC |
| *ACX3* | AAACCGATTAACAGCAGCATAGC | TATCAGTCTCACCAGCACAAGC |
| *ACX4* | AAGCAGAATGCTGAGAAAGACG | TTCGGTGAAAGGTTTGAATGAG |
