# Supplementary figures and images for: Melatonin-Stimulated Triacylglycerol Breakdown and Energy Turnover under Salinity Stress Contributes to the Maintenance of Plasma Membrane H+–ATPase Activity and K+/Na+ Homeostasis in Sweet Potato
Source: Front Plant Sci. 2018 Feb 27;9:256. doi: 10.3389/fpls.2018.00256 (PMC5835075; doi:10.3389/fpls.2018.00256)

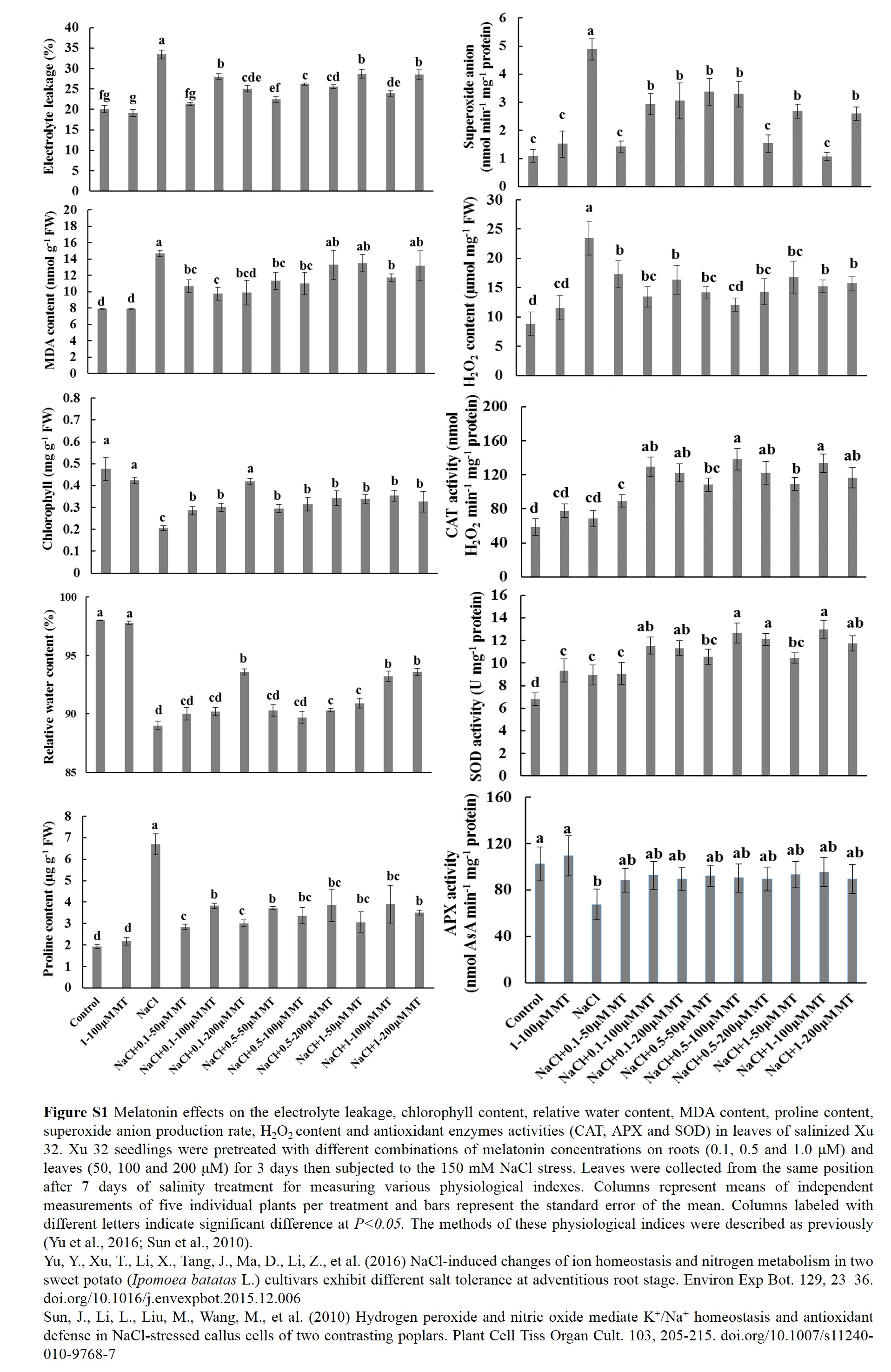

Supplement: Supplementary file 2 [file Image_1.JPEG]

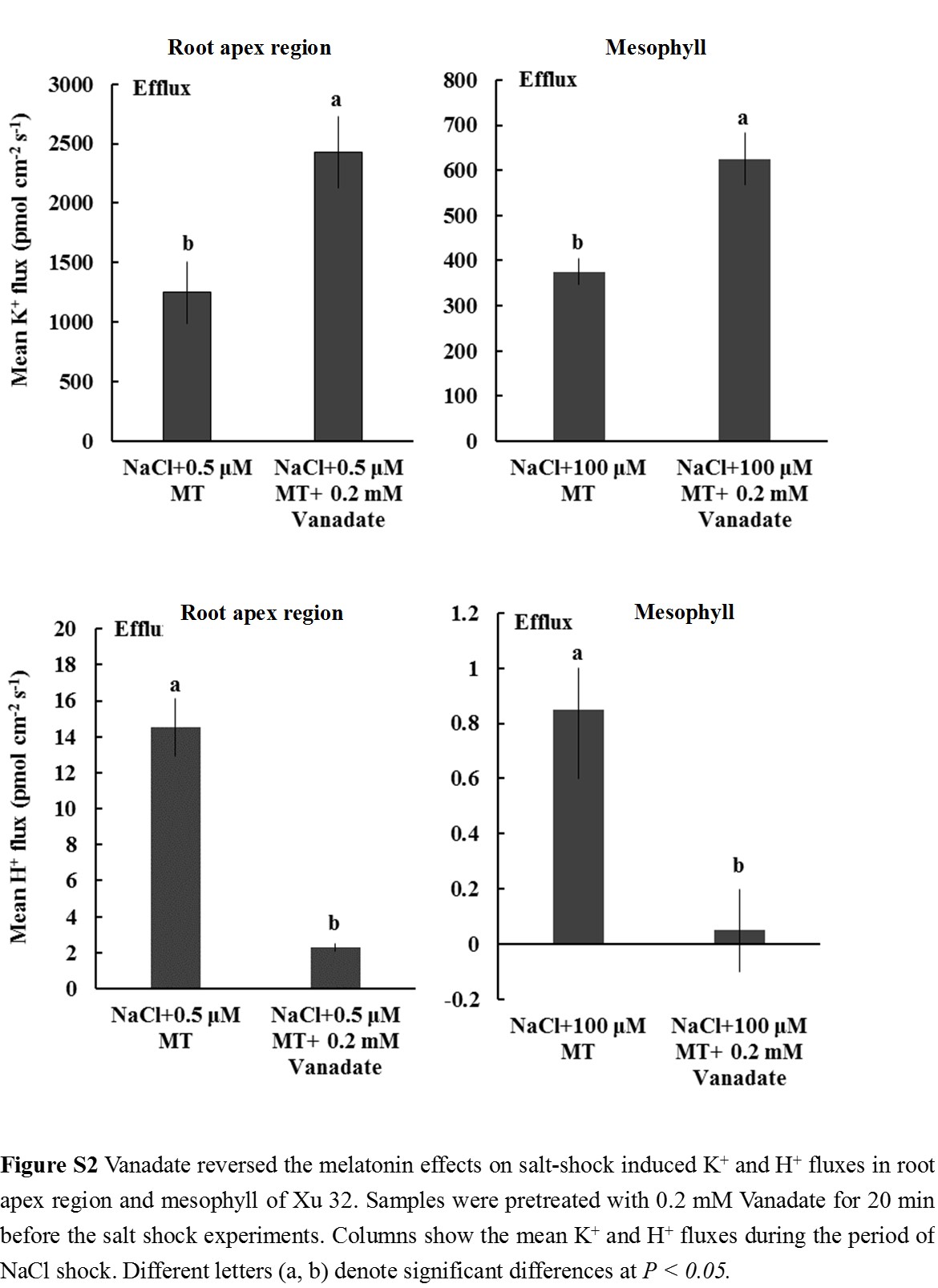

Supplement: Supplementary file 3 [file Image_2.JPEG]

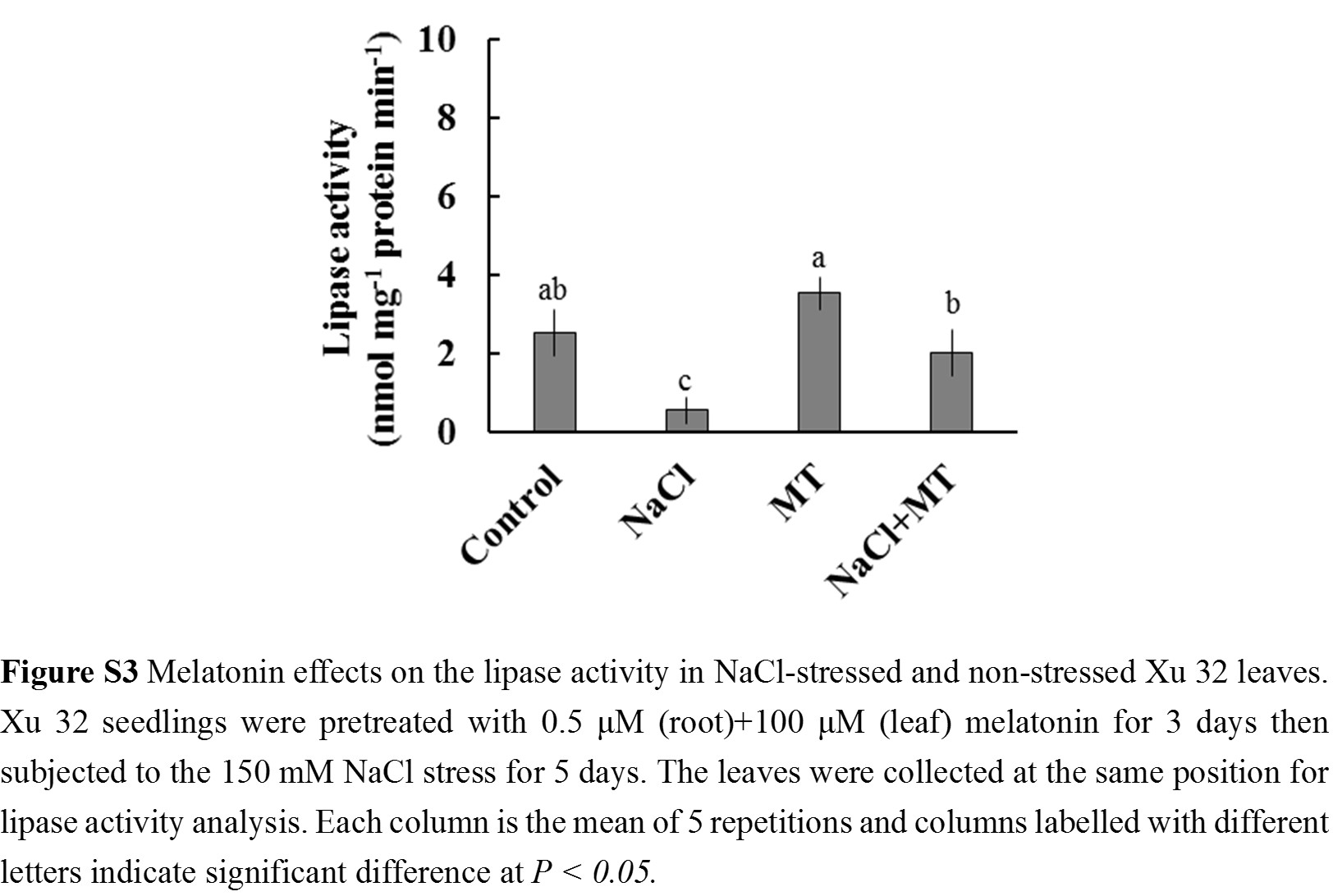

Supplement: Supplementary file 4 [file Image_3.JPEG]
